# Performance of brief ICF-sleep disorders and obesity core set in obstructive sleep apnea patients

**DOI:** 10.1186/s12931-020-01404-1

**Published:** 2020-06-22

**Authors:** Liang Xie, Qinhan Wu, Weiping Hu, Wenjing Li, Guiling Xiang, Shengyu Hao, Chengyao Guo, Hong Jiang, Xiaodan Wu, Xu Wu, Shanqun Li

**Affiliations:** 1 Department of Pulmonary Medicine, Zhongshan Hospital, Fudan University, 180 Fenglin Rd, Shanghai, China; 2 Clinical Centre for Sleep Breathing Disorders and Snoring, Zhongshan Hospital, Fudan University, Shanghai, China

**Keywords:** Obstructive sleep apnea, International classification of functioning, Disability and health, Brief ICF-sleep disorders Core set, Brief ICF-obesity Core set

## Abstract

**Background:**

Clinical questionnaires are mainly applied as screening tools for identification of the Obstructive sleep apnea (OSA) patients. Little attention has been paid to assess the body functions and health status of the patients. International Classification of Functioning, Disability and Health (ICF) was designed for better understanding and describing functioning and disability of patients. This study adopted the Brief ICF-Sleep Disorders and Obesity Core Set to evaluate the impairment of functioning and health status of OSA patients.

**Methods:**

Five hundred ninety-two participants were enrolled in this cross-sectional study. Data were collected using Brief ICF-Sleep Disorders and Obesity Core Set Polysomnography was performed and basic characteristics of the patients were recorded.

**Results:**

The scores for the component Body Functions and Code b130, b134, b140, b440, b530, s330, d160, d240, d450 of the two core sets were significantly different among the patients divided by apnea-hypopnea index (AHI) or oxygen saturation (SaO2) nadir, but the frequency of code s330, d160, d240, d450 was low. The Body Functions component of the both sets were closely related to neck circumference (NC), body mass index (BMI), apnea-hypopnea index (AHI) of the OSA patients. Body Functions of the Brief ICF-Sleep Disorders performed better with a threshold of 4 with sensitivity, specificity and area under the receiver operating characteristic curve (AUC) as 0.62, 0.74, 0.68(AHI ≥ 5), 0.69, 0.63, 0.66 (AHI ≥ 15), 0.75, 0.56, 0.66 (AHI ≥ 30), 0.56, 0.70, 0.63 (SaO2 nadir≤90%), 0.67, 0.66, 0.66 (SaO2 nadir<85%), 0.71, 0.59, 0.65 (SaO2 nadir<80%), separately.

**Conclusion:**

The Body Functions component of both two sets could be an evaluation tool of impairment of body functions for OSA patients. The Brief ICF-Sleep Disorders Body Functions component performed better with a threshold of 4 and might provide a new insight for physicians to assess OSA patients.

## Background

Obstructive sleep apnea (OSA) is a disorders that consists of recurrent obstruction of the upper airway during sleep, nocturnal hypoxemia, and excessive daytime sleepiness [1]. Untreated OSA could lead to increased mortality and morbidity [2]. Several studies have proved that OSA is closely associated with negative consequences such as diabetes, stroke, hypertension, neurocognitive dysfunction, and even the increased risks of traffic accidents [3–6]. The golden standard for diagnosing OSA is polysomnography (PSG) [7]. However, its high cost, relative inaccessibility and time consumption could delay the diagnosis and treatment of the OSA patients. What’s more, lack of awareness among physicians about the severity of patients could add difficulty to adjust the treatment without PSG. However, none of these questionnaires have investigated the impairment of functioning and health status of the patients.

The World Health Organization’s (WHO) International Classification of Functioning, Disability and Health (ICF) represents a comprehensive and universally common framework to understand and describe functioning and disability, by providing lists of essential categories that are related to specific health conditions and health care contexts [8]. Lots of studies have confirmed its validity in different diseases, including schizophrenia, asthma and chronic obstructive pulmonary disease (COPD) [9–11]. Also, ICF could be applied as a unifying model for the rehabilitation strategy [12]. A total of more than 1400 categories are included in the ICF classification, and several health conditions have been developed to facilitate its application, such as ICF Core Set for COPD, obesity, sleep disorders and diabetes, but there is no specific core set of OSA. Since OSA is a disease characteristic of obesity and sleep disorders, we adopted the ICF Core Set for Sleep Disorders and Obesity to evaluate patients’ functioning and health status and test validity of them. There are two versions of each ICF Core Set: the Brief and the Comprehensive Core Set. The Brief Core Set represents a minimum standard for collecting functioning data, while the Comprehensive one is supposed to represent the full spectrum of aspects of functioning and environmental factors relevant to the disease and is supposed to guide rehabilitation from a multidisciplinary perspective. Taking clinical practicability into consideration, we chose the Brief ICF Core Set of Sleep Disorders and Obesity for evaluation of patients’ functioning and health status.

The Brief ICF-Sleep Disorders Core Set consists 14 categories and 4 components, the latter include Body Functions and Structures, Activities and Participation and Environmental Factors (https://www.icf-research-branch.org/icf-core-sets-projects2/other-health-conditions/development-of-icf-core-sets-for-sleep). For Brief-ICF Obesity Brief Core Set, it concludes 8 categories and 3 components, the latter involve Body Functions, Activities and Participation and Environmental Factors (https://www.icf-research-branch.org/icf-core-sets-projects2/cardiovascular-and-respiratory-conditions). These two practical tools that cover the spectrum of symptoms and limitations in the functioning of all patients with sleep disorders or obesity, also considering the environments they live in.

As there was no investigation had confirmed the validity of the ICF Core Sets so far, this study aimed to validate Brief ICF-Sleep Disorders and Obesity Core Set in evaluating impairment of functioning and health status of OSA patients.

## Methods

### Patients and design

This nine-month cross-sectional study was carried out at Shanghai Zhongshan Hospital Affiliated to Fudan University from Feb 1st to Oct 31st, 2019. Adults aged over 18 years old who came to the respiratory clinic about OSA were enrolled. Exclusion criteria were as follows: (1) diagnosed OSA previously or accepted OSA treatment before; (2) acute or unstable medical or neurological conditions; (3) major psychiatric disorders or severe cognitive impairment.

Eventually 592 study subjects were enrolled and assessed by two ICF-based measurement tools, Brief ICF-Sleep Disorders and Obesity Core Set. Participants received an overnight polysomnography test (Alice-5 Respironics, Pittsburgh, Pennsylvania, USA). Based on the PSG tests, the subjects were classified by AHI or the lowest oxygen saturation (SaO2 nadir). In this study, the mean number of apneas and hypopneas per hour of sleep (apnea-hypopnea index (AHI))was calculated, and OSA was diagnosed if AHI was ≥5 events per hour [13]. Based on different values of AHI, the subjects were divided into no OSA group (AHI<5 events/h), mild OSA group (5 ≤ AHI<15 events/h), moderate OSA group (15 ≤ AHI<30 events/h) and severe OSA group (AHI ≥ 30 events/h). According to the level of oxygen saturation (SaO2 nadir), the participants could also be divided into group A (SaO2>90%), group B (85% ≤ SaO2 ≤ 90%), group C (80% ≤ SaO2<85%) and group D (SaO2<80%).

The Sleep Disorders Brief Core Set concludes 4 components and 14 codes, and the Obesity Brief Core Set consists of 8 codes and 3 components. By comparing every category and component of the two ICF core sets among different groups of participants classified by AHI or SaO2 nadir, we found that several categories and the Body Functions components showed potential as evaluating tools. Considering the frequency of these codes, only code b130, b134, b440 and b530 were suitable. Since these items belong to ICF-Body Functions components which represent the impairment of physiological and psychological functions of body systems, the Body Functions components could be suitable as an evaluation tool. Correlation of the Body Functions scores with each reported questionnaire and critical parameters of participants was analyzed separately to identify validation, and then the appropriate cut-off point was determined by comparing sensitivity, specificity, positive predictive value (PPV), negative predictive value (NPV), and area under the receiver operating characteristic curve (AUC).

**Fig. 1:**
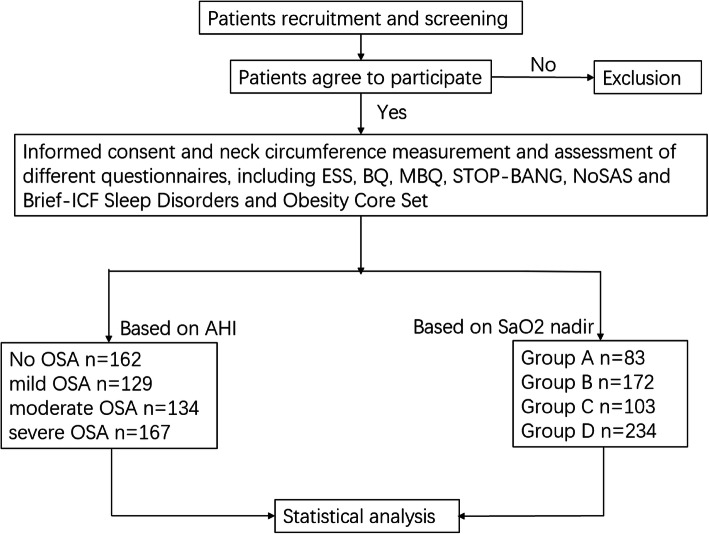
Flow diagram for participants in this study

The objective of the study and its requirements were explained to the subjects, and all participants provided written informed consent. This study received Ethical approval from the Zhongshan Hospital Institutional Review Board. The specific process can be referred to Fig. 1, and the sociodemographic and clinical characteristics of the participants are provided in Tables 1 and 2.

**Table 1:** Clinical characteristics of the study group

	Whole group (n/%)	No OSA (n/%)	OSA (n/%)	*p* value
Number	592/100	162/100	430/100	
**Men**	470/79.4	**100/61.7**	**370/86.0**	**< 0.0001**
**age**	49.21 ± 14.99	**44.67 ± 16.43**	**50.93 ± 14.04**	**0.0233**
**BMI [kg/m2]**	27.25 ± 4.66	**26.13 ± 4.42**	**27.58 ± 7.05**	**0.0016**
**Smoking**	263/44.4	**46/28.4**	**217/50.5**	**< 0.0001**
**Current smoker**	182/30.7	**31/19.1**	**151/35.1**	**0.0001**
Pack-years	19.91 ± 19.85	20.97 ± 23.92	19.69 ± 19.00	0.7435
**Quitting smoking**	93/15.7	**15/9.3**	**78/18.1**	**0.0077**
Quitting years	7.807 ± 7.716	6.643 ± 8.854	8.033 ± 7.524	0.4775
**NC**	39.47 ± 3.872	**37.49 ± 3.79**	**39.99 ± 3.82**	**< 0.0001**
Diabetes mellitus	87/14.7	17/10.5	70/16.3	0.0902
**Arterial hypertension**	268/45.3	**51/31.5**	**217/50.5**	**< 0.0001**
Short of breath	162/27.4	37/22.8	125/29.1	0.1479
**AHI**	21.00 ± 20.23	**1.713 ± 1.522**	**26.08 ± 19.70**	**< 0.0001**
**Max apnea time**	**43.31 ± 28.11**	**15.43 ± 17.28**	**53.99 ± 23.81**	**< 0.0001**
**SaO2 nadir**	**79.72 ± 10.52**	**88.19 ± 5.64**	**76.47 ± 10.15**	**< 0.0001**
Max pulse	109.7 ± 19.08	109.40 ± 20.47	109.8 ± 18.54	0.8531
**4% decline of SaO2 index**	**20.37 ± 19.06**	**5.04 ± 7.75**	**26.50 ± 18.79**	**< 0.0001**
rhinitis	149/25.2	40/24.7	109/25.3	0.9157
**pharyngitis**	127/21.5	**24/14.8**	**103/24.0**	**0.0181**
nasosinusitis	16/2.7	5/3.1	11/2.6	0.7772
nasal polyp	5/0.8	2/1.2	3/0.7	0.6180
polyp of vocal cord	4/0.7	3/1.9	1/0.2	0.0645
polypectomy	11/1.9	2/1.2	9/2.1	0.7356
Nasal septum deviation	6/1.0	1/0.6	5/1.2	1.00

Data are presented as Mean ± standard deviations. Differences were compared between no OSA and OSA groups

The data of significant difference were marked in bold.

*NC* neck circumference, *BMI* body mass index, *AHI* apnea-hypopnea index, *SaO2* oxygen saturation

**Table 2:** PSG examination parameters and questionnaire results of the participants

Category	No OSA(*n* = 162)	Mild (*n* = 129)	Moderate(*n* = 134)	Severe (*n* = 167)	*p* value
**AHI**	1.65 ± 1.54	9.55 ± 3.09	21.87 ± 4.33	48.21 ± 14.19	**< 0.0001**
**Max apnea time**	15.43 ± 17.28	36.31 ± 15.86	51.62 ± 18.84	69.35 ± 22.21	**< 0.0001**
**SaO2 nadir**	88.19 ± 5.64	82.76 ± 7.69	79.21 ± 7.98	69.96 ± 9.244	**< 0.0001**
Max pulse	109.40 ± 20.47	107.80 ± 18.55	109.60 ± 19.15	111.50 ± 18.02	0.25
**4% decline of SaO2 index**	5.04 ± 7.75	12.67 ± 7.40	21.41 ± 15.94	40.85 ± 16.9	**< 0.0001**

Data are presented as Mean ± standard deviations. Differences were compared among the 4 groups

SaO2, oxygen saturation. The data of significant difference were marked in bold

### Measures

In this study, the Brief ICF-Sleep Disorders and Obesity Core Sets were used to assess functioning and disability as well as environmental factors in all subjects. ICF qualifiers were applied to rate the degree of problems in each code of the Body Functions and Structures component and the Activity and Participation component with a generic five-point scale: no problem (0), mild problem (1), moderate problem (2), severe problem (3), and complete problem (4). Environmental factors were graded by three levels: being a facilitator (− 1), having no influence (0), or being a barrier (+ 1).

The relations of some critical parameters of the participants with ICF Core Set, including AHI, SaO2 nadir, NC and BMI were analyzed to evaluate the validity of the ICF Core Set as a measuring tool of severity of apnea, hypopnea and hypoxia.

### Data analysis

The characteristics of the subjects are presented as mean ± standard deviation for continuous variables, or number and percentage for categorical variables. Comparison between two groups was assessed by Student’s t test or Mann-Whitney rank sum test (for continuous variables) and Chi-square test or Fisher’s exact tests (for categorical variables). Comparison among multiple groups were assessed by one-way Analysis of Variance (ANOVA) or Kruskal-Wallis tests (for continuous variables) and Chi-square test or Fisher’s exact tests (for categorical variables). Regression analysis was used to determine the relationship between each one of AHI, BMI, NC, SaO2 nadir, and the two ICF-based measurement tools separately. Sensitivity, specificity, PPV, NPV and AUC of different cut-off versions of the Body Functions component scores were calculated including respective confidence intervals. Stata version 15 (Stata Corp, LLC, Texas, USA) and GraphPad Prism (version 6; GraphPad Software, San Diego, California, USA) were used for all statistical analysis and drawing graphs, and the level of significance was set to *p* <  0.05. The data of significant difference were marked in bold.

## Results

### Clinical characteristics, PSG examination and questionnaire results of the participants

Five hundred ninety-two participants were enrolled in this cross-sectional survey. According to PSG examination results, the subjects were divided into no OSA group (*n* = 162, AHI<5 events/h), mild OSA group (*n* = 129, 5 ≤ AHI<15 events/h), moderate OSA group (*n* = 134, 15 ≤ AHI<30 events/h), severe OSA group (*n* = 167, AHI ≥ 30 events/h) group based on the classification of AHI. Except max pulse, values of AHI, max apnea time, SaO2 nadir and 4% decline of SaO2 index were significantly different among the 4 groups.

### Differences of scores for each component of brief ICF-sleep disorders and obesity Core set among groups of participants

Severity of OSA can be measured by both AHI and lowest oxygen saturation (SaO2 nadir). According to the level of SaO2 nadir, the participants could also be divided into group A (*n* = 83, SaO2>90%), group B (*n* = 172, 85% ≤ SaO2 ≤ 90%), group C (*n* = 103, 80% ≤ SaO2<85%), group D (*n* = 234, SaO2<80%). The Sleep Disorders Brief Core Set concludes 4 components and the Obesity Core Set consists of 3 components. To identify whether the Brief ICF-Sleep and Obesity Core Sets could uncover the difference between participants divided by AHI and SaO2, the scores for each component of the two core sets were compared among the groups, by adding the scores of each category. When the subjects were classified by AHI, the Body Functions and Structures, Activities and Participation of Sleep Disorders Core Set and the Body Functions of Obesity Core Set could tell the difference among the groups (shown in Table 3). But only the scores for the Body Functions component of the two core sets were significantly different among the 4 groups classified by SaO2 nadir (shown in Table 4).

**Table 3:** Scores of Brief ICF-Sleep Disorder and Obesity Core Set in participants based on AHI classification

Category	No OSA(*n* = 162)	Mild (*n* = 129)	Moderate(*n* = 134)	Severe (*n* = 167)	*p* value
**ICF-Sleep Body Functions**	2.00 ± 1.58	3.30 ± 1.68	3.76 ± 2.09	4.68 ± 2.74	**< 0.0001**
**ICF-Sleep Body Structures**	0.23 ± 0.57	0.23 ± 0.60	0.41 ± 0.81	0.63 ± 1.11	**0.0010**
**ICF-Sleep Activities and Participation**	0.27 ± 1.00	0.23 ± 0.98	0.34 ± 1.07	0.51 ± 1.36	**0.0338**
ICF-Sleep Environmental Factors	−2.96 ± 0.25	− 2.98 ± 0.24	−2.94 ± 0.34	− 2.87 ± 0.50	0.1838
**ICF-Obesity Body Functions**	2.27 ± 1.23	2.58 ± 1.34	2.45 ± 1.11	3.01 ± 1.40	**< 0.0001**
ICF-Obesity Activities and Participation	0.38 ± 0.83	0.51 ± 1.18	0.79 ± 1.57	0.71 ± 1.52	0.1934
ICF-Obesity Environmental Factors	−1.98 ± 0.18	−1.98 ± 0.15	−1.96 ± 0.23	−1.95 ± 0.23	0.5767

Data are presented as Mean ± standard deviations. Differences were compared among the 4 groups. The data of significant difference were marked in bold

**Table 4:** Scores of Brief ICF-Sleep Disorder and Obesity Core Set in participants based on SaO2 classification

Category	A(*n* = 83)	B(*n* = 172)	C(*n* = 103)	D(*n* = 234)	*p* value
**ICF-Sleep Body Functions**	2.65 ± 1.69	3.25 ± 1.93	4.06 ± 2.14	4.80 ± 2.29	**< 0.0001**
ICF-Sleep Body Structures	0.08 ± 0.37	0.02 ± 0.12	0.03 ± 0.16	0.02 ± 0.13	0.47
ICF-Sleep Activities and Participation	0.29 ± 0.80	0.37 ± 1.22	0.35 ± 1.05	0.50 ± 1.27	0.47
ICF-Sleep Environmental Factors	0.046 ± 0.21	0.068 ± 0.31	0.075 ± 0.27	0.066 ± 0.29	0.89
**ICF-Obesity Body Functions**	2.05 ± 1.19	2.42 ± 1.26	2.61 ± 1.27	2.92 ± 1.31	**< 0.0001**
ICF-Obesity Activities and Participation	0.45 ± 1.16	0.74 ± 1.72	0.54 ± 1.22	0.76 ± 1.81	0.44
ICF-Obesity Environmental Factors	0.03 ± 0.17	0.04 ± 0.23	0.04 ± 0.19	0.03 ± 0.16	0.98

Data are presented as Mean ± standard deviations. Differences were compared among the 4 groups. The data of significant difference were marked in bold

### Frequency and extent of the participants’ impairments in different categories of sleep disorders and obesity brief Core set

The Sleep Disorders Brief Core Set concludes 14 codes. To further validate the performance of the categories, 592 participants were divided into 4 groups classified by AHI and SaO2 nadir, evaluating each category of the core set. As S-Tables 1 & 3 show, code b130, b134, b140, b440, s330, d160 and d240 display significant differences among subjects divided by AHI and SaO2 nadir, however, the positive rate of code s330, d160 and d240 was low and code b140 could not tell the difference when patients were classified by AHI. Compared with the other 3 parts, the categories of Body Functions showed great potential as an evaluation tool to assess the severity of the patients, and the more severe a patient was, the more likely his score was higher. There was no difference among the 4 groups of environmental factors part. Almost all the participants consider their family members, the health systems and policies as benefits.

The Obesity Brief Core Set involves 3 components and 8 codes, and it was applied to assess functioning and disability as well as environmental factors in 4 groups of participants based on AHI and SaO2 nadir classification (shown in S-Tables 2 & 4). The scores for code b130, b530, d240 and d450 were significantly different among the four groups of participants, but code d450 could not tell the difference when patients were divided by SaO2 nadir. Same as the Sleep Disorders Brief Core Set, there was no significant difference in the Environmental Factors component, and the frequency of code d240 and d450 was very low (seen in supplement materials, S-Tables 1, 2, 3 and 4).

### Potential for the body functions component of brief ICF-sleep disorders and obesity Core set as tools to evaluate body functioning of OSA patients

**Fig. 2:**
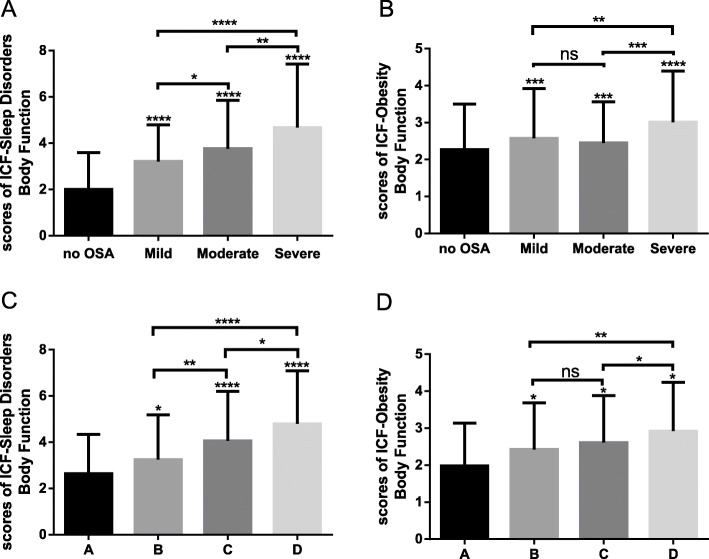
Sores of Body Functions component among different groups classified by AHI and SaO2 nadir. **a-b** Differences of scores of ICF-Sleep Disorder and Obesity Body Functions between each two groups of participants classified by AHI. **c-d** Differences of scores of ICF-Sleep Disorder and Obesity Body Functions between each two groups of participants classified by SaO2. Patients were classified by AHI in Figure **a-b**, by SaO2 in in Figure **c-d**. Statistical significant values are identified with *(α = 0.05). ****p < 0.0001, ***p < 0.001, **p < 0.01, *p < 0.05, ns, not significant

In Table 3, the parts of Body Functions and Structures, Activities and Participation of Brief ICF-Sleep Disorders Core Set show potential as an evaluation tool of body functioning and health status when the classification method was AHI. But when the patients were divided by SaO2 nadir, only Body Functions component worked (shown in Table 4). From S-Table 1 and 3, we find out that although the scores of some codes of the part Body Structures and Activities and Participation were significantly different among groups, the frequency was low. Taking all this into consideration, codes for Sleep Functions could be suitable as an evaluation tool of patients’ impairment of functioning. In the same way, the codes of Body Functions of Brief ICF-Obesity Core Set could be potential as a measuring tool, too. Then the scores of Body Functions of the two core sets were compared in patients divided by AHI and SaO2 nadir, which was shown in Fig. 2. Scores of Body Functions of each two groups of the participants were significantly different no matter the classification was by AHI or SaO2. However, the differences in mild and moderate groups or in Group B and C could not be recognized by Body Functions component of ICF-Obesity Core Set.

### Correlation of different parameters of participants with brief ICF-sleep disorders and obesity core set

Body mass index (BMI) and neck circumference (NC) are proved to be closely related to the morbidity and severity of OSA [14]. By comparing the correlation between BMI and NC and the Body Functions component scores of both core sets, we found significant relations. Then we analyzed the correlation between the Body Functions component and some critical parameters of PSG examination, including AHI and SaO2 nadir, and positive correlation was found (Table 5).

**Table 5:** Correlation of Participants’ different parameters with the Brief ICF-Sleep Disorder or Obesity Body Functions

Category	β^s^	95%CI^s^	β^o^	95%CI^o^
**AHI**	0.053	(0.044–0.063) ****	0.017	(0.011–0.023) ****
**BMI**	0.089	(0.041–0.14) ****	0.19	(0.17–0.21) ****
**NC**	0.10	(0.052–0.15) ****	0.13	(0.10–0.16) ****
**SaO2 nadir**	−0.073	(−0.0911 - -0.055) ****	−0.029	(−0.039 - -0.018) ****
**Brief ICF-Obesity or Sleep Disorder Body Functions scores**	0.86	(0.73–1.00) ****	0.30	(0.25–0.34) ****

Different superscript indicates different ICF core set. ^s^ means Brief ICF-Sleep Disorder Core Set Body Functions component. ^o^ means Brief ICF-Obesity Core Set Body Functions component. β means regression coefficient. CI means confidence interval

The data of significant difference were marked in bold

*NC* neck circumference, *SaO2* oxygen saturation

### Performance of the brief-ICF sleep disorders and obesity body functions in participants classified by AHI or SaO2

**Fig. 3:**
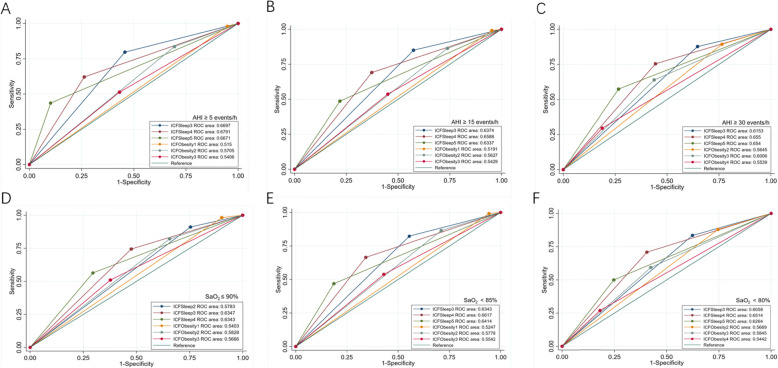
ROC curves for Body Functions component of Brief ICF-Sleep Disorders and Obesity Core Set. The area under the Receiver-operator characteristic (ROC) curve (AUC) closer to one indicates the higher evaluatiing accuracy of the screening questionnaire. **a**-**c** indicate AUC for an AHI cut-off≥5 and ≥ 15 and ≥ 30. **d**-**f** indicate AUC for an SaO2 cut-off≤90 and < 85% and < 80%

The values of sensitivity, specificity, PPV, NPV and AUC were calculated for all cut-off points (mild, moderate and severe OSA), considering AHI or SaO2 (shown in Tables 6 and 7 and Fig. 3). When the patients were classified by AHI, the threshold of 4 was suitable for Body Functions of ICF-Sleep Disorders with largest AUC and acceptable sensitivity and specificity, while the threshold of 3 of ICF-Obesity Core Set would be more suitable. As Table 6 shows, however the status of the patient changes, from normal to OSA, or mild to moderate, or moderate to severe, the ICF-Sleep Disorders Core Set with a threshold of 4 would be applicable, and whatever severity of hypoxia a patient is, the ICF-Sleep Disorders Core Set behaves better than the Obesity Core Set. When the participants were classified by SaO2, cut-off≥4 for ICF-Sleep Disorders Core Set and cut-off≥3 for ICF-Obesity Core Set might be suitable for evaluating severity of hypoxia, considering sensitivity, specificity and AUC. In general, the Body Functions component of the Brief ICF-Sleep Disorders Core Set with cut-off≥4 showed better sensitivity, specificity and AUC, and could be more reliable to assess the severity of apnea, hypopnea and hypoxia of OSA patient, as well as impairment of functioning.

**Table 6:** Predictive parameters of various Body Functions score cut-offs for different AHI levels

Cut-off points	Sensitivity	Specificity	PPV	NPV	AUC
AHI ≥ 5 events/h as the diagnostic criteria for OSA	Prevalence of OSA = 430/592 (72.64%)				
ICF-Sleep≥3	0.80	0.54	0.83	0.48	0.67 (0.62–0.72)
ICF-Sleep≥4	0.62	0.74	0.87	0.41	0.68 (0.63–0.73)
ICF-Sleep≥5	0.44	0.90	0.92	0.36	0.67 (0.63–0.71)
ICF-Obesity≥1	0.98	0.05	0.75	0.46	0.52 (0.49–0.54)
ICF-Obesity≥2	0.84	0.31	0.77	0.40	0.57 (0.52–0.62)
ICF-Obesity≥3	0.51	0.57	0.77	0.29	0.54 (0.49–0.59)
AHI ≥ 15 events/h as the diagnostic criteria for OSA	Prevalence of OSA =301/592 (50.84%)				
ICF-Sleep≥3	0.85	0.42	0.58	0.75	0.64 (0.60–0.68)
ICF-Sleep≥4	0.69	0.63	0.64	0.68	0.66 (0.62–0.70)
ICF-Sleep≥5	0.49	0.68	0.55	0.62	0.63 (0.59–0.68)
ICF-Obesity≥1	0.99	0.05	0.50	0.85	0.52 (0.50–0.53)
ICF-Obesity≥2	0.86	0.26	0.52	0.67	0.56 (0.53–0.60)
ICF-Obesity≥3	0.54	0.55	0.53	0.56	0.54 (0.50–0.59)
AHI ≥ 30 events/h as the diagnostic criteria for OSA	Prevalence of OSA = 167/592 (28.21%)				
ICF-Sleep≥3	0.88	0.35	0.33	0.89	0.62 (0.58–0.65)
ICF-Sleep≥4	0.75	0.56	0.38	0.86	0.66 (0.61–0.70)
ICF-Sleep≥5	0.57	0.73	0.44	0.82	0.65 (0.60–0.70)
ICF-Obesity≥2	0.89	0.24	0.30	0.86	0.56 (0.53–0.60)
ICF-Obesity≥3	0.64	0.56	0.35	0.81	0.60 (0.55–0.65)
ICF-Obesity≥4	0.30	0.81	0.37	0.76	0.55 (0.51–0.60)

*PPV* positive predictive value, *NPV* negative predictive value, *AUC* the area under the curve

**Table 7:** Predictive parameters of the various Body Functions score cut-offs for different SaO2 nadir levels

Cut-off points	Sensitivity	Specificity	PPV	NPV	AUC
SaO2 ≤ 90% as the diagnostic criteria for OSA	Prevalence of OSA = 509/592 (85.98%)				
ICF-Sleep≥2	0.91	0.25	0.89	0.30	0.58 (0.52–0.63)
ICF-Sleep≥3	0.59	0.39	0.86	0.13	0.63 (0.57–0.70)
ICF-Sleep≥4	0.56	0.70	0.92	0.20	0.63 (0.57–0.70)
ICF-Obesity≥1	0.98	0.10	0.88	0.46	0.54 (0.50–0.58)
ICF-Obesity≥2	0.82	0.34	0.89	0.23	0.58 (0.52–0.65)
ICF-Obesity≥3	0.51	0.62	0.90	0.17	0.57 (0.50–0.63)
SaO2 < 85% as the diagnostic criteria for OSA	Prevalence of OSA = 337/592 (56.93%)				
ICF-Sleep≥3	0.82	0.45	0.67	0.65	0.63 (0.59–0.68)
ICF-Sleep≥4	0.67	0.66	0.72	0.59	0.66 (0.62–0.71)
ICF-Sleep≥5	0.47	0.81	0.77	0.53	0.64 (0.60–0.68)
ICF-Obesity≥1	0.99	0.06	0.59	0.85	0.52 (0.51–0.54)
ICF-Obesity≥2	0.87	0.29	0.62	0.62	0.58 (0.54–0.62)
ICF-Obesity≥3	0.54	0.57	0.63	0.48	0.55 (0.51–0.60)
SAO2 < 80% as the diagnostic criteria for OSA	Prevalence of OSA =234 /592 (39.53%)				
ICF-Sleep≥3	0.84	0.74	0.81	0.77	0.61 (0.57–0.65)
ICF-Sleep≥4	0.71	0.59	0.54	0.75	0.65 (0.61–0.70)
ICF-Sleep≥5	0.50	0.75	0.58	0.69	0.63 (0.58–0.67)
ICF-Obesity≥2	0.88	0.25	0.44	0.76	0.57 (0.53–0.60)
ICF-Obesity≥3	0.59	0.58	0.48	0.68	0.58 (0.54–0.63)
ICF-Obesity≥4	0.27	0.82	0.50	0.63	0.54 (0.50–0.58)

*PPV* positive predictive value, *NPV* negative predictive value, *AUC* the area under the curve

## Discussion

Multiple studies have reported the application of the screening questionnaires in identifying OSA patients, such as ESS, BQ, MBQ, STOP-BANG and NoSAS questionnaires, however, little attention has been paid to evaluation of the functioning and health status of the OSA patients. OSA results in sleep fragmentation and recurrent hypoxemia and is associated with various adverse consequences that affect not only organ systems, but also quality of life [15]. Hence evaluation of functioning and disability of OSA patients is as important as diagnosis of the disease. ICF is a common framework for understanding and describing functioning and disability. Previous studies have reported that ICF cores sets offered recognized standards for assessing the functioning status of patients in various diseases, such as ADHD, schizophrenia and COPD, and played a critical role in rehabilitation [9, 16–18]. However, there is no specific ICF core set for OSA patients. The most relevant core sets that have been created were sleep disorder and obesity core sets. Using the sleep disorder and obesity ICF core set as guidelines, the given health status can be evaluated, and the management process of a patient can be assessed. Besides that, ICF Core Sets can also be used to rate the content validity of health status measures of the patients. The sleep disorder and obesity core sets were not created primarily for OSA, but the items of the core set could still be potential as evaluation tools for the patients. The aim of our investigation was to select the potential evaluation tools with the two coresets, and itis the first one to confirm the validity of Brief ICF-Sleep Disorders and Obesity Core Set in OSA patients in China.

Five hundred ninety-two participants were evaluated with Brief ICF-Sleep Disorders and Obesity Core Set. Most patients regard environmental factors as facilitators, including family and health systems. Though scores for Structures of Pharynx were significantly different between the groups, the frequency was low, so was the component Activities and Participation. Anatomical and non-anatomical factors are both critical causes of OSA [19]. Different from the anatomical factors, the non-anatomical factors, especially the tonicity of the pharyngeal dilators could not be evaluated by imaging, which resulting in the low detection of impairment of Structure of pharynx. Inability to maintain attention and vigilance is common reported in OSA patients [13], but the attentional deficiency is not common across all patients. It may occur in some, but not all OSA patients [20]. This could be the reason why the scores of code Attention functions and Focusing attention are much higher in the severe group and group D, but the exact number of patients who had the attention problems was low.

Since the significant codes with high frequency belong to the Body Functions component and the latter was statistically different among groups, this part was chosen as the measuring tool to evaluate the impairment of physiological and psychological functions. As an evaluation tool, specificity and sensitivity should be considered as well as AUC. The ICF-Sleep Disorders Body Functions component with a threshold of 4 proved to be applicable in assessing impairment of functioning of all patients. No matter what classification the patients were, divided by AHI or SaO2, or what cut-off the AHI or SaO2 was, cut-off≥4 has the largest AUC, more suitable sensitivity and specificity. This means whatever degree of apnea, hypopnea or hypoxia the patient was, the new evaluation method could be applicable to measure the impairment of body functions, or even help physicians adjust the patient’s treatment in time without PSG tests according to the scores, but all this need to be validated in the subsequent researches.

Compared with the Body Functions component of ICF-Obesity Core Set, the ICF-Sleep Disorders Body Functions has larger AUC, better sensitivity and specificity. Although obesity is an admitted risk factor for OSA [21], an overweight person did not certainly end up with OSA. Abdominal and neck fat accumulation seem to be more related to OSA than general obesity [22]. That could be reason why the ICF-Obesity Core Set could be applicable in evaluation, but the ICF-Sleep Disorders Core Set performed better.

Current clinical guideline applies AHI as the standard measurement of OSA [13]. However, OSA is a heterogeneous syndrome, influenced by varying risk factors, pathophysiological causes, clinical manifestations and consequences [23]. Hence, relying on AHI alone in diagnosis is not enough [24]. SaO2 nadir was shown to be closely related to concentration of high-sensitivity C-reactive protein, and lower SaO2 nadir could lead to higher proinflammatory markers [25]. In the meantime, previous studies mostly concentrate on the performance of questionnaires in subjects classified by AHI, little attention has been paid to the validity of questionnaires in patients with different level of SaO2 nadir. Our research validated the Brief ICF Core Set in patients classified by AHI as well as SaO2 nadir. The results show that the Body Functions component proved to be applicable in assessing impairment of functioning in patients with various degrees of AHI and SaO2 nadir. Meanwhile, the body functions component of the ICF core set positively corelated with AHI and SaO2 nadir and was significantly different among groups, which means it would be useful for measuring the severity of apnea, hypopnea and hypoxia. In summary, the Body Functions component was proved to be applicable in evaluating the impairment of body functions, and closely relate to severity of apnea, hypopnea and hypoxia. But to confirm the threshold of 4 of the Brief ICF-Sleep Disorders Core Set as a follow-up indicator, more researches of a larger sample need to be done.

## Conclusions

This research is the first one to investigate the performance of the Brief ICF-Sleep Disorders and Obesity Core Set in OSA patients classified by AHI and SaO2 nadir. It reveals the Body Functions component of Brief ICF-Sleep Disorders Core Set closely related to the degree of apnea, hypopnea and hypoxia, and could be applicable in evaluation of impairment of functioning and health status with a threshold of 4, This approach provides a new insight for physicians to evaluate OSA patients and offers an evaluation tool to assess the functioning and health status of them.

## Supplementary information

**Additional file 1.** S-Table 1 Measuring impairments of the participants classified by AHI with Brief ICF-Sleep Disorders Core Set.

**Additional file 2.** S-Table 2 Measuring impairments of the participants classified by AHI with Brief ICF-Obesity Core Set.

**Additional file 3.** S-Table 3 Measuring impairments of the participants classified by SaO2 nadir with Brief ICF-Sleep Disorders Core Set.

**Additional file 4.** S-Table 4 Measuring impairments of the participants classified by SaO2 nadir with Brief ICF-Obesity Core Set.

## Data Availability

The datasets used and analyzed during the current study are available from the corresponding author on reasonable request.

## Abbreviations

OSA: Obstructive sleep apnea
ICF: International Classification of Functioning, Disability and Health
WHO: World Health Organization
NC: Neck circumference
BMI: Body mass index
AHI: Apnea-hypopnea index
COPD: Chronic obstructive pulmonary disease
ANOVA: Analysis of Variance
SaO2: Oxygen saturation
PPV: Positive predictive value
NPV: Negative predictive value
AUC: Area under the receiver operating characteristic curve
ROC: Receiver-operator characteristic
