# Supplementary material for: Performance of brief ICF-sleep disorders and obesity core set in obstructive sleep apnea patients
Source: Respir Res. 2020 Jun 22;21:156. doi: 10.1186/s12931-020-01404-1 (PMC7310139; doi:10.1186/s12931-020-01404-1)
Supplement: Supplementary file 1 — Additional file 1. S-Table 1 Measuring impairments of the participants classified by AHI with Brief ICF-Sleep Disorders Core Set. [file 12931_2020_1404_MOESM1_ESM.docx]

**S-Table 1** Measuring impairments of the participants classified by AHI with Brief ICF-Sleep Disorders Core Set

| Code | Category title | No OSA (n=162) | | | Mild (n=129) | | | Moderate(n=134) | | | Severe (n=167) | | | p value |
| --- | --- | --- | --- | --- | --- | --- | --- | --- | --- | --- | --- | --- | --- | --- |
|  |  | n | % |  | n | % |  | n | % |  | n | % |  |  |
| **Body Functions** | |  |  |  |  |  |  |  |  |  |  |  |  |  |
| b110 | Consciousness functions | 0 | 0.00 | 0 | 1 | 0.78 | 0.0077±0.0078 | 1 | 0.75 | 0.015±0.17 | 2 | 1.20 | 0.03±0.28 | 0.61 |
| **b130** | **Energy and drive functions** | 70 | 43.21 | 0.73±0.97 | 122 | 94.57 | 1.46±0.080 | 120 | 89.55 | 1.57±1.00 | 148 | 88.62 | 1.84±1.06 | **< 0.0001** |
| **b134** | **Sleep functions** | 43 | 26.54 | 0.40±0.72 | 62 | 48.06 | 0.75±1.00 | 62 | 46.27 | 0.80±1.01 | 83 | 49.70 | 0.86±1.07 | **< 0.000**1 |
| b140 | Attention functions | 8 | 4.94 | 0.093±0.47 | 3 | 2.33 | 0.023±0.015 | 8 | 5.97 | 0.060±0.24 | 16 | 9.58 | 0.16±0.54 | 0.06 |
| **b440** | **Respiration functions** | 69 | 42.59 | 0.78±1.07 | 82 | 63.57 | 1.08±0.0.98 | 92 | 68.66 | 1.33±1.09 | 127 | 76.05 | 1.80±1.26 | **< 0.000**1 |
| **Body Structures** | |  |  |  |  |  |  |  |  |  |  |  |  |  |
| s110 | Structure of brain | 4 | 2.47 | 0.031±0.21 | 0 | 0.00 | 0 | 2 | 1.49 | 0.01±0.12 | 4 | 2.40 | 0.02±0.15 | 0.34 |
| **s330** | **Structure of pharynx** | 24 | 14.81 | 0.19±0.43 | 21 | 16.28 | 0.22±0.60 | 34 | 25.37 | 0.39±0.80 | 48 | 28.74 | 0.61±1.11 | **0.0025** |
| s430 | Structure of respiratory system | 1 | 0.62 | 0.012±0.16 | 1 | 0.78 | 0.010±0.09 | 1 | 0.75 | 0.01±0.09 | 0 | 0.00 | 0 | 0.75 |
| **Activities and Participation** | |  |  |  |  |  |  |  |  |  |  |  |  |  |
| **d160** | **Focusing attention** | 3 | 1.85 | 0.08±0.37 | 6 | 4.65 | 0.040±0.32 | 8 | 5.97 | 0.060±0.24 | 21 | 12.57 | 0.17±0.49 | **0.026** |
| **d240** | **Handling stress and other psychological demands** | 9 | 5.56 | 0.09±0.47 | 11 | 8.53 | 0.06±0.39 | 10 | 7.46 | 0.14±0.67 | 24 | 14.37 | 0.17±0.62 | **0.040** |
| d475 | Driving | 9 | 5.56 | 0.093±0.43 | 11 | 8.53 | 0.12±0.54 | 10 | 7.46 | 0.14±0.59 | 18 | 10.78 | 0.17±0.58 | 0.38 |
| **Environmental Factors** | |  |  |  |  |  |  |  |  |  |  |  |  |  |
| e310 | Immediate family | 2 | 0.23 | 1.01±0.11 | 2 | 1.55 | 1.02±0.20 | 1 | 0.75 | 1.01±0.09 | 5 | 2.99 | 1.04±0.25 | 0.45 |
| e355 | Health professionals | 2 | 0.23 | 1.01±0.11 | 3 | 2.33 | 1.03±0.21 | 5 | 3.73 | 1.05±0.28 | 7 | 4.19 | 1.06±0.32 | 0.37 |
| e580 | Health services, systems and policies | 1 | 0.62 | 1.01±0.08 | 0 | 0.00 | 1 | 0 | 0.00 | 1 | 2 | 1.20 | 1.02±0.17 | 0.40 |

Data are presented as Mean±standard deviations. Differences were compared among the 4 groups. The data of significant difference were marked in bold.
