# Supplementary material for: Performance of brief ICF-sleep disorders and obesity core set in obstructive sleep apnea patients
Source: Respir Res. 2020 Jun 22;21:156. doi: 10.1186/s12931-020-01404-1 (PMC7310139; doi:10.1186/s12931-020-01404-1)
Supplement: Supplementary file 2 — Additional file 2. S-Table 2 Measuring impairments of the participants classified by AHI with Brief ICF-Obesity Core Set. [file 12931_2020_1404_MOESM2_ESM.docx]

**S-Table 2**Measuring impairments of the participants classified by AHI with Brief ICF-Obesity Core Set

| Code | Category title | No OSA (n=162) | | | Mild (n=129) | | | Moderate (n=134) | | | Severe (n=167) | | | p value |
| --- | --- | --- | --- | --- | --- | --- | --- | --- | --- | --- | --- | --- | --- | --- |
|  |  | n | % |  | n | % |  | n | % |  | n | % |  |  |
| **Body Functions** | |  |  |  |  |  |  |  |  |  |  |  |  |  |
| **b130** | **Energy and drive functions** | 70 | 43.21 | 0.73±0.97 | 122 | 94.57 | 1.43±0.80 | 120 | 89.55 | 1.57±1.00 | 148 | 88.62 | 1.84±1.06 | **< 0.0001** |
| **b530** | **Weight maintenance functions** | 94 | 58.02 | 0.75±0.86 | 92 | 71.32 | 0.88±0.83 | 94 | 70.15 | 0.83±0.78 | 154 | 92.22 | 1.21±0.84 | **< 0.0001** |
| **Activities and Participation** | |  |  |  |  |  |  |  |  |  |  |  |  |  |
| **d240** | **Handling stress and other psychological demands** | 9 | 5.56 | 0.09±0.47 | 11 | 8.53 | 0.06±0.39 | 10 | 7.46 | 0.14±0.67 | 24 | 14.37 | 0.17±0.62 | **0.040** |
| **d450** | **Walking (G)** | 1 | 0.62 | 0.01±0.08 | 2 | 1.55 | 0.02±0.12 | 8 | 5.97 | 0.07±0.31 | 8 | 4.79 | 0.08±0.38 | **0.025** |
| d455 | Moving around (G) | 29 | 17.90 | 0.25±0.59 | 28 | 21.71 | 0.36±0.83 | 35 | 26.12 | 0.51±1.01 | 38 | 22.75 | 0.37±0.78 | 0.27 |
| d570 | Looking after one's health | 5 | 3.09 | 0.04±0.22 | 5 | 3.88 | 0.07±0.40 | 6 | 4.48 | 0.07±0.35 | 10 | 5.99 | 0.09±0.38 | 0.62 |
| **Environmental Factors** | |  |  |  |  |  |  |  |  |  |  |  |  |  |
| e110 | Products or substances for personal consumption | 1 | 0.62 | 1.01±0.08 | 1 | 0.78 | 1.02±0.18 | 1 | 0.75 | 1.01±0.17 | 1 | 0.60 | 1.01±0.08 | 0.99  0.45 |
| e310 | Immediate family | 2 | 0.23 | 1.01±0.11 | 2 | 1.55 | 1.02±0.20 | 1 | 0.75 | 1.01±0.09 | 5 | 2.99 | 1.04±0.25 |  |

Data are presented as Mean±standard deviations. Differences were compared among the 4 groups. The data of significant difference were marked in bold.
