# Supplementary material for: Performance of brief ICF-sleep disorders and obesity core set in obstructive sleep apnea patients
Source: Respir Res. 2020 Jun 22;21:156. doi: 10.1186/s12931-020-01404-1 (PMC7310139; doi:10.1186/s12931-020-01404-1)
Supplement: Supplementary file 3 — Additional file 3. S-Table 3 Measuring impairments of the participants classified by SaO2 nadir with Brief ICF-Sleep Disorders Core Set. [file 12931_2020_1404_MOESM3_ESM.docx]

**S-Table 3** Measuring impairments of the participants classified by SaO2 nadir with Brief ICF-Sleep Disorders Core Set

| Code | Category title | A(n=83) | | | B(n=172) | | | C(n=103) | | | D(n=234) | | | p value |
| --- | --- | --- | --- | --- | --- | --- | --- | --- | --- | --- | --- | --- | --- | --- |
|  |  | n | % |  | n | % |  | n | % |  | n | % |  |  |
| **Body Functions** | |  |  |  |  |  |  |  |  |  |  |  |  |  |
| b110 | Consciousness functions | 0 | 0.00 | 0 | 0 | 0.00 | 0 | 1 | 0.97 | 0.01±0.11 | 3 | 1.28 | 0.03±0.22 | 0.37 |
| **b130** | **Energy and drive functions** | 54 | 65.06 | 1.02±0.74 | 134 | 77.91 | 1.60±0.91 | 84 | 81.55 | 1.81±0.96 | 188 | 80.34 | 1.88±0.97 | **< 0.0001** |
| **b134** | **Sleep functions** | 25 | 30.12 | 0.45±0.69 | 76 | 44.19 | 0.86±0.98 | 40 | 38.83 | 0.79±0.98 | 109 | 46.58 | 0.95±1.06 | **0.0039** |
| **b140** | **Attention functions** | 3 | 3.61 | 0.03±0.17 | 5 | 2.91 | 0.02±0.12 | 5 | 4.85 | 0.04±0.19 | 22 | 9.40 | 0.15±0.47 | **0.0057** |
| **b440** | **Respiration functions** | 25 | 30.12 | 0.40±0.77 | 94 | 54.65 | 0.94±0.94 | 73 | 70.87 | 1.35±0.96 | 178 | 76.07 | 1.93±1.13 | **< 0.0001** |
| **Body Structures** | |  |  |  |  |  |  |  |  |  |  |  |  |  |
| s110 | Structure of brain | 0 | 0.00 | 0 | 3 | 1.74 | 0.02±0.19 | 2 | 1.94 | 0.01±0.11 | 5 | 2.14 | 0.02±0.15 | 0.66 |
| **s330** | **Structure of pharynx** | 18 | 21.69 | 0.26±0.44 | 37 | 21.51 | 0.27±0.44 | 22 | 21.36 | 0.36±0.48 | 50 | 21.37 | 0.23±0.43 | **0.22** |
| s430 | Structure of respiratory system | 0 | 0.00 | 0 | 1 | 0.58 | 0.02±0.17 | 2 | 1.94 | 0.03±0.16 | 0 | 0.00 | 0 | 0.12 |
| **Activities and Participation** | |  |  |  |  |  |  |  |  |  |  |  |  |  |
| **d160** | **Focusing attention** | 4 | 4.82 | 0.05±0.21 | 6 | 3.49 | 0.07±0.39 | 6 | 5.83 | 0.06±0.24 | 22 | 9.40 | 0.15±0.42 | **0.04** |
| **d240** | **Handling stress and other psychological demands** | 5 | 6.02 | 0.11±0.24 | 14 | 8.14 | 0.08±0.41 | 10 | 9.71 | 0.15±0.59 | 25 | 10.68 | 0.24±0.51 | **< 0.0001** |
| d475 | Driving | 1 | 1.20 | 0.02±0.12 | 16 | 9.30 | 0.17±0.55 | 8 | 7.77 | 0.19±0.71 | 23 | 9.83 | 0.19±0.61 | 0.13 |
| **Environmental Factors** | |  |  |  |  |  |  |  |  |  |  |  |  |  |
| e310 | Immediate family | 1 | 1.20 | 0.02±0.12 | 3 | 1.74 | 0.02±0.15 | 2 | 1.94 | 0.03±0.16 | 4 | 1.71 | 0.02±0.15 | 0.98 |
| e355 | Health professionals | 2 | 2.41 | 0.03±0.17 | 5 | 2.91 | 0.04±0.19 | 3 | 2.91 | 0.04±0.19 | 7 | 2.99 | 0.04±0.19 | 0.99 |
| e580 | Health services, systems and policies | 0 | 0.00 | 0 | 1 | 0.58 | 0.01±0.09 | 1 | 0.97 | 0.01±0.11 | 1 | 0.43 | 0.01±0.07 | 0.82 |

Data are presented as Mean±standard deviations. Differences were compared among the 4 groups. The data of significant difference were marked in bold.
