# Supplementary material for: Performance of brief ICF-sleep disorders and obesity core set in obstructive sleep apnea patients
Source: Respir Res. 2020 Jun 22;21:156. doi: 10.1186/s12931-020-01404-1 (PMC7310139; doi:10.1186/s12931-020-01404-1)
Supplement: Supplementary file 4 — Additional file 4. S-Table 4 Measuring impairments of the participants classified by SaO2 nadir with Brief ICF-Obesity Core Set. [file 12931_2020_1404_MOESM4_ESM.docx]

**S-Table 4** Measuring impairments of the participants classified by SaO2 nadir with Brief ICF-Obesity Core Set

| Code | Category title | A(n=83) | | | B(n=172) | | | C(n=103) | | | D(n=234) | | | P value |
| --- | --- | --- | --- | --- | --- | --- | --- | --- | --- | --- | --- | --- | --- | --- |
|  |  | n | (%) |  | n | (%) |  | n | (%) |  | n | (%) |  |  |
| **Body Functions** | |  |  |  |  |  |  |  |  |  |  |  |  |  |
| **b130** | **Energy and drive functions** | 54 | 65.06 | 1.02±0.74 | 134 | 77.91 | 1.60±0.91 | 84 | 81.55 | 1.81±0.96 | 188 | 80.34 | 1.88±0.97 | < 0.0001 |
| **b530** | **Weight maintenance functions** | 58 | 69.88 | 0.49±0.54 | 127 | 73.84 | 0.83±0.86 | 77 | 74.76 | 1.04±0.93 | 172 | 73.50 | 1.17±0.86 | **< 0.0001** |
| **Activities and Participation** | |  |  |  |  |  |  |  |  |  |  |  |  |  |
| **d240** | Handling stress and other psychological demands | 5 | 6.02 | 0.11±0.24 | 14 | 8.14 | 0.08±0.41 | 10 | 9.71 | 0.15±0.59 | 25 | 10.68 | 0.24±0.51 | **< 0.0001** |
| d450 | Walking (G) | 2 | 2.41 | 0.02±0.12 | 3 | 1.74 | 0.02±0.12 | 4 | 3.88 | 0.03±0.16 | 10 | 4.27 | 0.08±0.39 | 0.26 |
| d455 | Moving around (G) | 15 | 18.07 | 0.20±0.44 | 37 | 21.51 | 0.04±0.87 | 21 | 20.39 | 0.33±0.72 | 57 | 24.36 | 0.46±0.97 | 0.41 |
| d570 | Looking after one's health | 4 | 4.82 | 0.06±0.24 | 5 | 2.91 | 0.02±0.1s5 | 3 | 2.91 | 0.05±0.35 | 14 | 5.98 | 0.11±0.43 | 0.20 |
| **Environmental Factors** | |  |  |  |  |  |  |  |  |  |  |  |  |  |
| e110 | Products or substances for personal consumption | 1 | 1.20 | 0.01±0.08 | 1 | 0.58 | 0.02±0.18 | 1 | 0.97 | 0.01±0.11 | 1 | 0.43 | 0.01±0.08 | 0.99 |
| e310 | Immediate family | 1 | 1.20 | 0.02±0.12 | 3 | 1.74 | 0.02±0.15 | 2 | 1.94 | 0.03±0.16 | 4 | 1.71 | 0.02±0.15 | 0.98 |

Data are presented as Mean±standard deviations. Differences were compared among the 4 groups. The data of significant difference were marked in bold.
